# Physiochemically and Genetically Engineered Bacteria: Instructive Design Principles and Diverse Applications

**DOI:** 10.1002/advs.202403156

**Published:** 2024-06-12

**Authors:** Xia Lin, Rong Jiao, Haowen Cui, Xuebing Yan, Kun Zhang

**Affiliations:** ^1^ Central Laboratory and Department of Ultrasound Sichuan Academy of Medical Sciences Sichuan Provincial People's Hospital School of Medicine University of Electronic Science and Technology of China No. 32, West Second Section, First Ring Road Chengdu Sichuan 610072 China; ^2^ Department of Oncology Affiliated Hospital of Yangzhou University. No.368 Hanjiang Road, Hanjiang District Yangzhou Jiangsu Province 225012 China

**Keywords:** bacterial derivatives, design principles, diverse applications, genetically‐engineered bacteria, physiochemically‐engineered bacteria

## Abstract

With the comprehensive understanding of microorganisms and the rapid advances of physiochemical engineering and bioengineering technologies, scientists are advancing rationally‐engineered bacteria as emerging drugs for treating various diseases in clinical disease management. Engineered bacteria specifically refer to advanced physiochemical or genetic technologies in combination with cutting edge nanotechnology or physical technologies, which have been validated to play significant roles in lysing tumors, regulating immunity, influencing the metabolic pathways, etc. However, there has no specific reviews that concurrently cover physiochemically‐ and genetically‐engineered bacteria and their derivatives yet, let alone their distinctive design principles and various functions and applications. Herein, the applications of physiochemically and genetically‐engineered bacteria, and classify and discuss significant breakthroughs with an emphasis on their specific design principles and engineering methods objective to different specific uses and diseases beyond cancer is described. The combined strategies for developing in vivo biotherapeutic agents based on these physiochemically‐ and genetically‐engineered bacteria or bacterial derivatives, and elucidated how they repress cancer and other diseases is also underlined. Additionally, the challenges faced by clinical translation and the future development directions are discussed. This review is expected to provide an overall impression on physiochemically‐ and genetically‐engineered bacteria and enlighten more researchers.

## Graphical abstract

Engineered bacteria specifically refer to advanced physiochemical or genetic modifications of diagnostic bacteria, which can compensate for the shortcomings of natural strains. After engineering, these diagnostic bacteria are encouraged to lyse tumors, regulate immunity and cellular communication such immune cells, influence metabolic pathways, withstand harsh environments, efficiently deliver cargos, reduce virulence and express exogenous proteins or molecular biosynthetic species, etc.

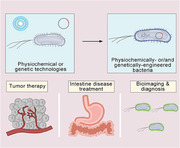

## 1 Introduction

Bacterial‐represented microorganisms emerge in ancient times, and earlier identified as the primary crime culprit of many infection‐related diseases or chronic inflammatory diseases,^[^ ^1^, ^2^, ^3^ ^]^ which have aroused increasing interests in developing antimicrobial materials, drugs, dressings and technologies.^[^ ^4^, ^5^, ^6^ ^]^ As the modern medicine advances, bacterial and their components have exhibited widespread advantageous aspects inducing drug manufacturing factory, CRISPR/Cas9 gene editing and normal physiological activities regulation.^[^ ^7^, ^8^, ^9^ ^]^ As the current hot spot and star, gut microbiota have been found to regulate various diseases such as inflammatory bowel disease (IBD), tumor,^[^ ^10^, ^11^ ^]^ autism, etc.^[^ ^12^, ^13^, ^14^ ^]^ Typically, scientists believe that dysbiosis of the gut microbiome is inextricably connected to the development of cancer,^[^ ^15^, ^16^, ^17^ ^]^ and microbiome can have a significant impact on circulating nutrients and metabolic homeostasis through the production or transformation of neurotransmitters, short‐chain fatty acids, amino acids and secondary bile acids.^[^ ^17^, ^18^ ^]^ All of these determine that gut microbiota can be regarded as targets to engineer various drugs, materials or technologies.

Apart from negatively serving as the target, bacterial and their components are serve as treatment tools or platforms,^[^ ^19^ ^]^ which are able to effectively inhibit the progression of diseases, and is gradually becoming a powerful clinical weapon in the fight against disease such as oncolytic bacteria.^[^ ^20^, ^21^, ^22^, ^23^ ^]^ Especially, the in‐depth and comprehensive understanding of gut microorganisms and their functional roles in human health and disease progression beneficially develop these specific bacterial‐targeting drugs and other regulation technologies.^[^ ^24^, ^25^ ^]^ Since the pioneering work underscoring injected inactivated bacteria into the body to activate immune cells to inhibit the development of malignant disease;^[^ ^26^ ^]^ more bacteria‐mediated disease treatments have been developed and highlighted, and explorations on the design and application of bacterial‐mediated treatment objective to diseases is booming.^[^ ^27^, ^28^ ^]^ Typically, anaerobic bacteria or facultative anaerobes bacteria can also be used as drug carriers to targeted deliver anti‐tumor drugs into solid tumor since they spontaneously tend to enter hypoxic and necrotic tumor regions, which enables the deeper penetration into tumor tissues and improves drug concentration and distribution range.^[^ ^22^, ^29^, ^30^, ^31^, ^32^, ^33^, ^34^ ^]^ Intriguingly, bacteria as the components of in situ tumor vaccinations to stimulate immune responses have aroused an research surge.^[^ ^35^, ^36^ ^]^

To realize the therapeutic intention, physiochemical interface modification or/and specific genetically‐edited target expression of bacterial is indispensable. Bacteria can be physiochemically and genetically engineered with technological tools to compensate for the shortcomings of natural strains as therapeutic agents.^[^ ^23^, ^37^ ^]^ Physiochemical engineering can provide a cost‐effective and easy‐to‐implement method to modify bacteria without disrupting their structure and intrinsic antitumor effects,^[^ ^38^, ^39^ ^]^ endowing bacteria with stronger abilities to withstand harsh environments and deliver cargos efficiently.^[^ ^40^ ^]^ Genetically engineering can be leveraged to design genetic circuits through gene editing and pave an avenue to manipulate bacteria including reducing virulence, controlling behavior, expressing targeted exogenous proteins or molecular biosynthetic species and enhancing interactions between bacteria and organismal immune cells and/or tumor cells.^[^ ^41^, ^42^, ^43^ ^]^ Currently, genetically‐engineered bacteria provided a powerful platform where they could remove bacterial virulence genes and construct nutrient deficient strain for modifying tumor‐specific bacteria^[^ ^41^ ^]^ and balancing gut microbial homeostasis.^[^ ^11^ ^]^

With the significant advances in synthetic biology,^[^ ^23^, ^44^ ^]^ biomaterials,^[^ ^45^, ^46^, ^47^ ^]^ and emerging therapeutic modalities such as photodynamic therapy (PDT),^[^ ^48^ ^]^ thermal photothermal therapy (PTT),^[^ ^49^ ^]^ sonodynamic therapy (SDT)^[^ ^50^ ^]^ and radiotherapy (RT),^[^ ^51^ ^]^ etc.,^[^ ^52^, ^53^, ^54^ ^]^ researchers are enriching the applications of physiochemically‐ and genetically‐engineered bacteria in combination with other therapeutic modalities.^[^ ^55^, ^56^ ^]^ These armed physiochemically‐ and genetically‐engineered bacteria can not only improve disease‐targeted treatments, but also provide diverse tools or strategies for diseases prediction, diagnostic,^[^ ^57^ ^]^ and medical imaging.^[^ ^58^, ^59^ ^]^ There are only two theranostic bacteria‐related reviews that focused on different aspects. One review underlined the comparisons between nanomedicine and bacterial in surface physiochemical properties, delivery manner and challenges, several application cases of nanomedicine‐bacterial combined therapy.^[^ ^60^ ^]^ Another review highlighted the anti‐tumor applications of genetically‐engineered bacteria including directly killing tumor cells, activating anti‐tumor immune responses, wherein the multiple combined theranostic modalities especially with nanomedicine were emphasized.^[^ ^41^ ^]^

**Figure 1:**
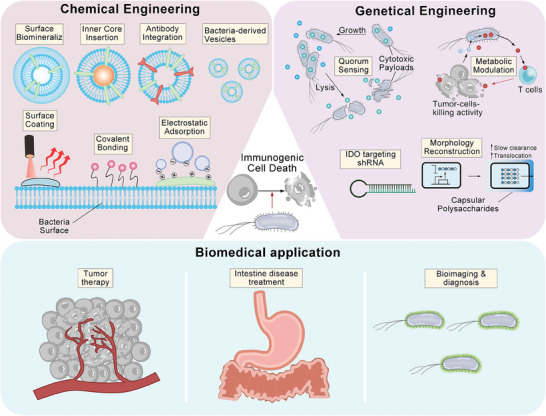
Schematic illustration of design strategies and disease treatment strategies for chemically and genetically engineered bacteria.

However, there are almost no comprehensive and detailed reviews that concurrently cover physiochemically‐ & genetically‐engineered bacteria and their derivatives including their personal engineering methods and classifications, let alone the distinctive and inspiring design principles and the engineering‐oriented various functions and applications. In this review, we first gave a systematic and generalized impression on physiochemically‐ and genetically‐ engineered bacteria. Subsequently, we classified and discussed relevantly significant advances in physiochemical engineering and genetic engineering of bacteria including their derivatives as biotherapeutic agents with an emphasis on their appealing and specific design principles and engineering methods objective to different specific uses and diseases beyond cancer. We also underlined the combined strategies in the development of in vivo biotherapeutic agents based on these physiochemically‐ and genetically‐engineered bacteria or bacterial derivatives, and elucidated how they acted on cancer and other diseases (**Figure** **1**). In the end, the current hurdles and concerns of clinical translation were proposed, and corresponding potential solutions and future development directions were provided. This review made a comprehensive outline and discussion on physiochemically‐ and genetically‐engineered bacteria, and provided an insight into this the cutting‐edge spot, which would attract more attentions and enlighten researchers.

## 2 Physiochemically‐Engineered Bacteria

Physiochemical engineering provides a low‐cost and easy strategy to alter the physicochemical and biochemical properties of bacterial and their derivatives such as surface groups, functions, surface charge, proteins, hydrophobicity, etc.^[^ ^61^, ^62^ ^]^ Physiochemically‐engineered techniques specifically refer to the use of nanomaterials and therapeutic agents or molecules to assembly on bacterial surfaces via various interactions including covalent binding, self‐assembly, electrostatic adsorption, biotic‐abiotic system interactions, and bacterial membrane encapsulation (**Table** **1**).^[^ ^63^, ^64^ ^]^ The aims of physiochemically‐engineered bacteria or derivatives were summarized into four aspects: i) modifying the bacterial interface to resist unfavorable survival environments and protect the stabilizing activity of biologics; ii) carrying therapeutic agents to function as in vivo delivery vectors; iii) enhancing specific adhesion to target tissues of the organism; iv) targeting immune cells or the disease microenvironment to stimulate organismal immune modulation.

**Table 1:** Representative paradigms of physiochemically‐engineered bacteria with different design principles.

Design strategies	Name	Type	References
Interface physiochemically‐engineered strategies	*Escherichia coli* Nissel 1917 (EcN)	Covalent binding	[65]
	*Escherichia coli* (Gram‐) and *Staphyloccocus epidermidis* (Gram+)	Click electrochemistry reaction	[66]
	*Bifidobacterium longum* probiotics	Artificial enzyme modification	[68]
	*Lactobacillus rhamnosus* GG	Self‐assembly	[70]
	*Bacteroides fragilis* (BF839)	Electrostatic adsorption	[73]
Physiochemically‐modified bacterial derivatives	*Streptococcus pneumoniae*	Capsular polysaccharides‐coated gallium‐polyphenol nanoparticles	[82]
	*Akkermansia muciniphila*	Bacterial outer membrane vesicles	[83]
Physiochemical engineering for specific tissue or organ adhesion	*Escherichia coli Nissel* 1917 (EcN)	Famultifunctional nanocoating	[88]
	*Candida albicans*	Modulating the physical form fungal mannans	[89]
Physiochemical engineering for specific tumor microenvironment regulation	*Proteus mirabilis* (A‐gyo) and *Rhodopseudomonas palustris* (UN‐gyo)	Isolation from solid tumor biopsies	[91]
Design strategies for combined physical therapy	*Escherichia coli* MG1655	Bacterial biohybrid microrobotic	[94]

The bacterial interface modification has been witnessed to trigger specific physiological signals and reactions. Given that, researchers can change the biological interface characteristics to impart bacteria with more physiological functions including adhesion, proliferation and differentiation, which is expected to strengthen the interaction between bacteria and the surrounding environment.

### 2.1 Interface Physiochemically‐Engineered Strategies for Bacteria

#### 2.1.1 Covalent Binding on Bacterial Surfaces

**Figure 2:**
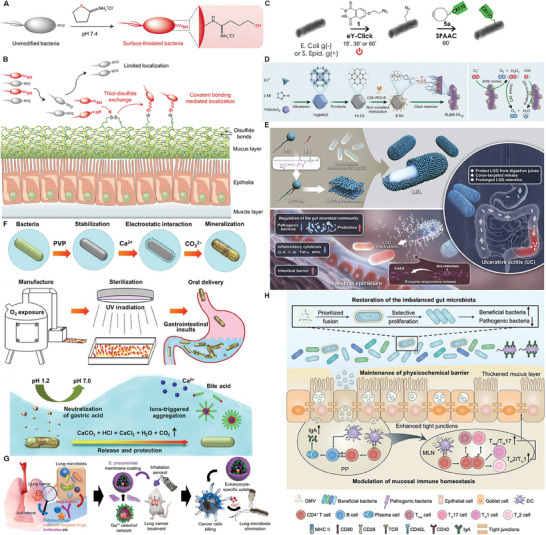
Physicochemically‐engineered strategies for designer bacterial interfaces and bacterial derivatives. A) Schematic diagram of preparation of surface sulfhydryl bacteria B) Schematic illustration of chemical reaction‐mediated covalent localization of bacteria. Reproduced with permission.^[^ ^65^ ^]^ Copyright 2022, Springer Nature. C) Schematic illustration of electrochemical coating of bacterial surfaces. Reproduced with permission.^[^ ^66^ ^]^ Copyright 2023, Springer Nature. D) Schematic diagram of preparation of artificial‐enzyme‐armed probiotics and its simulation of SOD and CAT antioxidant enzymes. Reproduced with permission.^[^ ^68^ ^]^ Copyright 2023, Springer Nature. E) Schematic illustration of the LBL preparation process and the mechanism of LBL treatment of ulcerative colitis. Reproduced with permission.^[^ ^70^ ^]^ Copyright 2022, John Wiley and Sons. F) Schematic illustration of biointerface mineralization that generates ultraresistant gut microbes. Reproduced with permission.^[^ ^73^ ^]^ Copyright 2023, AAAS. G) Schematic diagram of improving cancer chemotherapy by consuming lung microflora with bioinspired nanomedicine. Reproduced with permission.^[^ ^82^ ^]^ Copyright 2023, John Wiley and Sons. H) Schematic diagram of OMV from symbiotic bacteria can regulate versatility in intestinal homeostasis through membrane fusion. Reproduced with permission.^[^ ^83^ ^]^ Copyright 2023, AAAS.

Chemical bonding that connected functional groups, functional molecules or nanoparticles with bacteria is the most prevalent despite the presence of potential function alteration risk because it can directly switch the physiochemical properties of bacteria and arm them with more new functions. Chemical bonding is stable and avoid shedding of modified molecules or nanoparticles, and current bonding reactions cover a wide range, including amine‐reactive, thiol‐reactive and carboxyl‐reactive to form covalent bonds in situ on bacterial surfaces. Consequently, the covalent binding promotes probiotic adhesion to the gut and a balanced microecological environment. As a typical paradigm, Luo reported that the amino groups on the surface of many bacteria could be transformed into free thiols under cytocompatible conditions by iminothiolane cyclolytic reaction,^[^ ^65^ ^]^ and then the thiolated live bacteria on the surface created new disulfide bonds by thiol‐disulfide exchange. Afterwards, these new disulfide bonds were further evolved into covalent bonds with polymer‐containing proteins on the surface of tissues in vivo to promote the colonization of the mucus layer, which significantly relieved the symptoms of jejunal mucositis (**Figure** **2**A,B).

Click electrochemistry reaction is a remarkable route to realize the biosynthesis of functional agents via the covalent bonding linkage, which is not confined to the in vivo or cellular levels, but also can be expanded to the in vitro reaction. Click‐electrochemistry under exogenously‐applied electric field is one of the click reactions. In a recently reported study, G. Gouin and co‐workers used the simple click‐electrochemistry means to label bacteria as well as other organisms such as viruses and cells.^[^ ^66^ ^]^ In detail, they applied electric potential to a buffer containing the mixture of *N*‐methylluminol and bacteria, and made them chemically crosslink, where *N*‐methylluminol served as the anchoring group that could bind with azide‐functionalized tyrosine‐selective protein on bacterial and other organisms via the electrochemical stimuli‐triggered one‐electron oxidation. More significantly, this process was rapid and lossless without structure destruction and infectivity impairment, and failed to alter the abilities of bacterial survival and replication and expansion. This electrochemistry‐ignited click coupling could be also extended as an external biological method to modify bacteria with ligands or probes, similar to metabolic oligosaccharide engineering method (Figure 2C).

Biotin‐avidin interaction points out another direction to develop chemical modification strategies, Jia et al established a general tyrosinase‐catalysed oxidative coupling reaction (TyOCR) strategy, which was harnessed to realize bacterial surface modification and conjugate with specific molecules or nanoparticles.^[^ ^64^ ^]^ The modification of bacterial surfaces with functional coatings retained bacterial activity and simultaneously fulfilled the special role of synthetic materials, and it has led to the development of biotic‐abiotic hybrid systems.^[^ ^67^ ^]^ Chen and co‐workers modified an artificial enzyme containing a metal active canter (i.e., Fe‐based signal atoms (SAs)) via a boronic acid‐poly(ethylene glycol) (C18‐PEG‐B) linkage on the *Bifidobacterium longum* (BL) probiotic surface to obtain BL@B‐SA50 (Figure 2D).^[^ ^68^ ^]^ Fe‐based SAs with strong antioxidant capacity rapidly scavenged reactive oxygen species (ROS) in inflammatory environment, which in turn protected BL probiotic from oxidative damages, effectively alleviating IBD and repairing the intestinal immune system. With the help of physiochemically‐engineered strategies, the existing groups on the surface of living bacteria are directly modified to form covalent bonds. Thus, the physiochemical engineering allows these engineered bacteria to retain the parent physiological characteristics of biological agents and effectively regulate the interface interactions between biological agents and hosts.

#### 2.1.2 Self‐Assembly on Bacterial Surfaces

In recent years, liposomes have great application prospects as drug carriers, which are mainly composed of phospholipids and cholesterol, while phospholipids are the building blocks of lipid bilayer membranes, which share low toxicity and high biocompatibility and feature approximately identical structure to biological membranes.^[^ ^69^ ^]^ As a result, the self‐assembly driving force, i.e., hydrophobicity interaction, is expected to make these liposomes‐based nanoparticles self‐assemble on bacterial membrane via the membrane fusion strategy. The most typical case was made by Wu and co‐workers,^[^ ^70^ ^]^ where they designed phospholipids with one end tethered to a colon‐targeted drug, i.e., 5‐aminosalicylic acid (5‐ASA) prodrug‐balsalazide (Bal) and another end conjugated with bacteria surface to synthesize LPC‐Bal (LBL). These phospholipids could self‐assemble by non‐covalent bonding forces between supra‐molecules modified on the surface of Lactobacillus rhamnosus GG to construct an engineered probiotic (LBL) (Figure 2E). Interestingly, phospholipids not only provided protection for the probiotic against gastrointestinal digestive juices and made probiotic remain active in this study, but also carried the Bal prodrug. After oral administration, LBL released 5‐ASA in the presence of colonic bacterial azo‐reductases, which significantly reduced the expression of IL‐6 and IL‐1β and TNF‐α and myeloperoxidase (MPO), effectively inhibited ulcerative colitis and enhanced the colonization of LGGs, thereby acting as a regulator of the intestinal microflora. Apart from phospholipids, hydrogels could also be coated onto bacteria and serve as a protection layer to resist harsh conditions, e.g., A chitosan‐modified sodium alginate hydrogels as a “protective membrane” was coated to protect bacteria,^[^ ^71^ ^]^ which enhanced their tolerance in the strong acidic environment of gastrointestinal tract. After rational design and function prediction, physicochemical engineering utilized intermolecular interactions to achieve the self‐assembly of nanomaterials, and then grafted with bacterial surfaces to construct innovative biologics with multifunctional drug‐coating effects. The self‐assembly process and subsequent engineering not only minimized the impacts of physiochemical engineering on bacterial activity, but also conferred bacteria with robust resistance to environmental threats and invasion. This strategy provides an avenue to precisely manipulate bacteria function at the nanoscale level, promising high potential applications in biomedicine.

#### 2.1.3 Electrostatic Adsorption on Bacterial Surfaces

Biological agents are inevitably exposed to oxygen, ultraviolet light and alcohol during preparation, production or storage. Physiochemical engineering has been also documented to enhance the resistance of bacteria to adverse environments in vivo or in vitro.^[^ ^72^ ^]^ Inspired by the biomimetic mineralization of biointerfaces, Geng and co‐workers used polyvinyl‐pyrrolidone as a stabilizer to uniformly modify *Bacteroides fragilis* (BF839),^[^ ^73^ ^]^ which could promote the binding of calcium ions on the surface of bacteria. Ultimately, the carbonate binding through electrostatic interaction shaped into a calcium carbonate coating on the surface of bacteria (Figure 2F). It is worth mentioning that the electrostatic adsorption and subsequent biomineralization coatings on bacteria not only improved the bioavailability, but also released the bacteria by neutralizing gastric acid after oral administration. Coincidently, the generated calcium ions could further induce the aggregation of bile acid micelles to protect the bacteria from the repetitive damages by gastric acid and bile acid, which provided a new route to produce oral formulations of in vivo biopharmaceuticals.

As indicated in above statements, weak intermolecular interaction including electrostatic effect, hydrophobic effect, π‐π conjugation and other intermolecular forces also provided a similar “adhesive” effect that acted like in‐situ chemical reaction to provide a seed for the physiochemical engineering of bacteria and promote them to more accurately localize the target tissue. The modified functional coatings served as a “protective film” to protect bacteria from the harsh surrounding environment, maintain vitality and play a therapeutic effect on the disease.^[^ ^38^, ^74^, ^75^ ^]^ As a typical paradigm, Cao group constructed lipid membrane‐coated bacteria (LCB) via biointerfacial supramolecular self‐assembly after vortexing for 15 min.^[^ ^76^ ^]^ LCB survivability remained virtually unchanged even after experiencing treatments with strong acids, strong bases, and simulated gastric and intestinal fluids, which were highly preferable for disease treatment. Such bacterial multifunctional coatings based on electrostatic interaction not only improved in vivo utilization efficiency of drugs, but also enhanced oral drug compliance in clinical patients, during which the physiological properties of bacteria remained intact without impairment.

### 2.2 Design Strategies for Physiochemically‐Modified Bacterial Derivatives

Beyond physiochemical and genetical‐engineered bacteria, the biocompatibility, immunostimulatory and specific tropism properties of bacteria are also broadening the applications of bacterial derivatives or metabolites.^[^ ^77^ ^]^ There are also many strategies for designing the encapsulation and delivery vehicles of drugs using bacterial derivatives.^[^ ^19^ ^]^ It is no exaggeration to say that anaerobic bacterial hypoxic targeting and nutrition chemotaxis are the most design rationales of almost all engineered bacteria‐based delivery system. Based on them, engineered bacteria can serve as self‐navigating micro‐/nano‐robot to target and retain in tumor due targeting their hypoxia microenvironment.^[^ ^78^ ^]^

It has been widely documented that physiochemical engineering techniques combined with nanomaterials carrying therapeutic molecules collaborated together to exert anti‐tumor, anti‐viral, and anti‐infective effects.^[^ ^15^, ^79^, ^80^, ^81^ ^]^ Typically, Zhang et al found that the symbiotic flora in the lungs reduced the efficacy of chemotherapeutic drugs through natural transformation.^[^ ^82^ ^]^ Therein, they extracted the capsular polysaccharides (CPs) from *Streptococcus pneumoniae* and then used them to encapsulate GaTa nanoparticles which are self‐assembly from gallium ions (Ga^3+^) and Tannic acid (Ta), and successfully constructed inhalable CPs‐coated gallium‐polyphenol (GaTa‐CP) nanoparticles (NPs). Similar to the “Trojan horse” strategy, CPs are able to masquerade as normal host tissues and reduce the clearance of GaTa‐CP NPs by macrophages. In addition, etoposide was encapsulated with GaTa‐CP NPs to form GaTa‐CP@Eto NPs. The release of Ga^3+^ in tumor site can destroy iron metabolism of bacteria, release etoposide and enhance cytotoxicity to lung cancer cells (Figure 2G).

Bacterial outer membrane vesicles (OMVs) are spherical lipid bilayer nanoparticles secreted by Gram‐negative bacteria. The nano‐size of OMVs can be enriched in the tumor site by the enhanced permeability and retention effect and it contains a large number of pathogen‐associated molecular patterns (PAMPs), which can be recognized by host immune system and trigger immune response. In the latest findings,^[^ ^83^ ^]^ OMVs derived from *Akkermansia muciniphila* (AKK) could select beneficial intestinal flora for membrane fusion, especially *Lactobacillus spp*, and thus restore intestinal homeostasis. It could be also translocated into Peyer's patches, and induce mucosal immunoglobulin A (IgA) responses, and modulate cellular immunity in mesenteric lymph nodes to promote intestinal homeostasis (Figure 2H). Further, oral administration of Akk‐OMVs maintained intestinal homeostasis and enhanced the efficacy of anti‐programmed cell death protein 1 (PD‐1) in the treatment of colorectal cancer in a mouse model induced by dextrose sulfate sodium. In addition, bacterial ghosts that were characterized as empty membranes of Gram‐negative bacteria retained a greater degree of bacterial immunogenicity, but had no bacterial activity, which determine the high safety as delivery vehicles or vaccine adjuvants.^[^ ^84^ ^]^

Through mimicking the structure of bacterial components or derivatives, some biomimetic nanostructures were also empowered to play the roles of the imitated components or derivatives. Inspired by the filamentous morphology of bacterial flagella, Yin et al developed polydiiododiacetylene (PIDA) nanofibers,^[^ ^85^ ^]^ which could orally deliver computed tomography contrast agent. Specifically, PIDA was synthesized by topological chemical polymerization, which not only adhered to the mucus layer of gastrointestinal tract but also localized the inflamed lesions for a long time after oral administration. More importantly, PIDA could be specifically discerned by computed tomography imaging and illuminate the contour of the gastrointestinal wall, which, thereby, united with ROS scavenging to reduce inflammation and rebalance the intestinal ecology.

### 2.3 Physiochemical Engineering for Specific Tissue or Organ Adhesion

**Figure 3:**
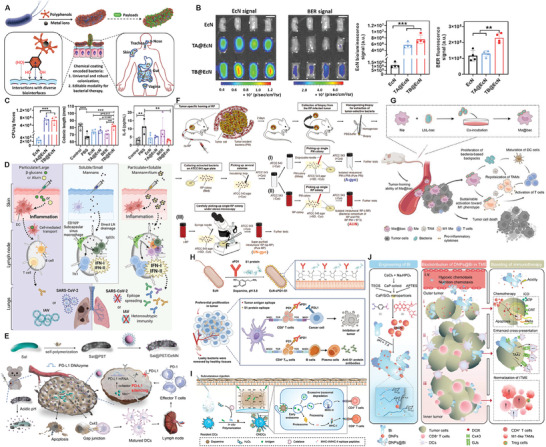
Physiochemically‐engineered bacteria for specific tissue or organ adhesion and specific tumor microenvironment regulation. A) Schematic diagram of preparation of coated bacteria and its interaction with host. B) Schematic diagram of EcN bioluminescence and BER fluorescence and their corresponding fluorescence intensity. C) Schematic diagram of EcN count in stool samples, average colon length after different treatments and K level of IL‐6 in serum. Reproduced with permission.^[^ ^88^ ^]^ Copyright 2023, Elsevier. D) Schematic diagram of adjuvant characteristics for improving immune stimulation by regulating the physical properties of fungal mannan. Reproduced with permission.^[^ ^89^ ^]^ Copyright 2022, Cell Press. E) Schematic for illustrating the construction of Sal@PST/DzMN, and its function mechanisms in tumor tissue for enhanced immunotherapy. Reproduced with permission.^[^ ^90^ ^]^ Copyright 2023, John Wiley and Sons. F) Schematic illustration of isolation of extremely effective anticancer bacteria from solid tumors. Reproduced with permission.^[^ ^91^ ^]^ Copyright 2023, John Wiley and Sons. G) Preparation of Mø@bac and regulation of tumor immunosuppressive microenvironment mediated by Mø@bac. Reproduced with permission.^[^ ^28^ ^]^ Copyright 2023, John Wiley and Sons. H) Schematic diagram on how dressed EcN with a hybrid immunoactive surface and induced antiviral and anticancer double immune responses. Reproduced with permission.^[^ ^1^ ^]^ Copyright 2023, John Wiley and Sons. I) Schematic illustration of in situ polymerization‐mediated antigen presentation. Reproduced with permission.^[^ ^92^ ^]^ Copyright 2023, American Chemical Society. J) Schematic illustration of enhanced antigen presentation of tumors with engineered Bi for chemo‐immunotherapy. Reproduced with permission.^[^ ^93^ ^]^ Copyright 2023, American Chemical Society.

Chemical engineering strategies can be used to modulate bacteria to enhance interactions at the tissue interface and increase the ability to actively target organs or colonize the gut. The scientific community now recognizes that gut homeostasis affects the immune system and human metabolism, and regulating gut bacteria could treat obesity, diabetes, infectious diseases, chronic diseases and cancer.^[^ ^11^ ^]^ In addition to providing essential nutrients to the host through metabolic activities, the microbiota is capable of synthesizing and secreting a wide range of functional signaling molecules, such as neurotransmitters and hormone‐like substances. These functional signaling molecules are able to directly or indirectly regulate the immune response of host, and which are essential for maintaining tissue homeostasis.^[^ ^86^, ^87^ ^]^ Physiochemically‐engineered bacteria or derivatives could provide further therapeutic strategies for balancing gut homeostasis. Fe^III^ and TA could self‐assemble with each other via coordination to form TA/Fe^III^ nanofilms with excellent biocompatibility on bacteria, and this self‐assembly process was disabled to affect the viability and metabolism of bacteria. In the report, Luo et al used FeCl_3_ and TA sequentially to mix with *Escherichia coli* Nissel 1917 (EcN) by vortex stirring,^[^ ^88^ ^]^ and a nanocoating (TA@EcN) was obtained on the bacterial surface within 30 seconds (**Figure** **3**A). Interestingly, the pyrocatechol/catechol moiety on the TA/Fe^III^ film has strong adhesion strength, which prompted TA@EcN to not only stably colonize the biointerfaces of murine and porcine including the mucosa of the skin, intestines, vagina, trachea, and nose, but also adhere to drugs such as berberine (BER) and doxorubicin (DOX) and exert therapeutic effects. In a dextran sulfate sodium‐induced mouse colitis model, oral TB@EcN (TA@EcN loading BER) for intestinal colonization was significantly increased and synergistically enhanced the treatment effect of colitis (Figure 3B,C).

In another study, the physical properties (e.g., size and solubility) of regulatory pattern recognition receptors (PRRs) ligands were found to determine the immune responses.^[^ ^89^ ^]^ The researchers isolated soluble mannans from *Candida albicans*, and validated that although these soluble mannans failed to cause skin inflammation when injected intradermally, they induced drainage lymph node amplification and lymphocyte aggregation, thereby eliciting an earlier and more pronounced immunostimulatory responses than particulate β‐glucans. This phenomenon was primarily attributed to the upregulations of mannans‐arise type I and II IFN pathway. Especially, SARS‐CoV‐2 Spike protein and mannans formulated with alum generated anti‐Spike type 1 immunity and neutralizing antibodies with broad epitope specificity (Figure 3D).

Although bacteria‐based tumor therapy could be strengthened by virtue of their tumor‐specific active tropism, the rapid systemic clearance, non‐specific distribution and neutrophils‐mediated immune restriction inevitably discouraged their outcome. Aiming at these concerns, an engineered *Salmonella* that behaved as carriers to electrostatically adsorb DNAzyme (Dz)‐modified MnO_2_ nanoparticles (DzMN) was coated with polyserotonin (PST) via the oxidation and self‐polymerization of PST serotonin monomer. PST coating resisted the rapid systemic clearance of bacteria and meanwhile maintained the active tropism to tumor. After degradation and shedding of PST in acidic tumor microenvironment, *Salmonella* and the hitchhiked DzMN exerted their personal anti‐tumor actions including immune activation and PD‐L1 knockdown to boost anti‐tumor immunotherapy(Figure 3E).^[^ ^90^ ^]^

### 2.4 Physiochemical Engineering for Specific Tumor Microenvironment Regulation

Physiochemically‐engineered bacteria and derivatives l immunotherapy strategies can pinpoint the tumor environment and improve the safety and efficacy of bacterial therapies. More and more studies have shown that there are intratumoral bacteria in most solid tumors, some of which are natural and effective tumor‐specific bacteria, which preferentially grow and proliferate in tumor tissues to lyse tumor and simultaneously encourage immune cells to infiltrate into tumors.^[^ ^11^ ^]^ For example, Goto et al.^[^ ^91^ ^]^ isolated *Rhodopseudomonas palustris* (RP)‐associated intratumoral lysogenic bacteria from colon cancer tissues, e.g., A‐gyo, UN‐gyo, and AUN. In mouse tumor models, a single injection could trigger long‐lasting anti‐tumor immunity, where mice after intravenous injection of AUN or A‐gyo received 100% survival after 120 days, exhibiting a significantly‐prolonged survival rate (Figure 3F). In addition, the use of natural immunogenicity of bacteria to stimulate adoptively infused macrophages is another pathway to enhance anti‐tumor immunotherapy. Zhang et al. successively bond chitosan (CHI) and carboxymethyl chitosan onto *Escherichia coli* Nissle 1917 (EcN) to form an adhesive nano‐coating,^[^ ^28^ ^]^ which facilitated the binding of bacteria to macrophages (Mø@bac). Mø@bac migrated into the tumor microenvironment (TME) within a short period of time, and bacterial proinflammatory factors enabled the sustained activation of Mø@bac toward the M1 phenotype, and concurrently facilitated the repolarization of endogenous tumor‐associated macrophages (TAMs) to remodel the immunosuppressive TME (Figure 3G).

There are many other experiences and strategies in favor of tumor microenvironment regulations to enhance engineered bacteria‐based anti‐tumor efficacy. Inspired by the fact that nanoparticular polydopamine (PDA) surfaces can bind different functional groups (e.g., catechols and amines), Liu et al.^[^ ^1^ ^]^ covalently linked nucleophilic groups from the immune checkpoint inhibitor α‐PD‐1 and the novel coronavirus SARS‐CoV‐2 spike 1 (S1) protein to PDA by Michael addition and Schiff base reaction to form a hybrid immunoreactive surface. The in situ polymerized PDA was then deposited onto the bacterial EcN surface by forming covalent or hydrogen bonds, termed EcN‐αPD1‐S1 (Figure 3H). Engineered bacterial EcN‐αPD1‐S1 could penetrate deeply into tumor tissues to enhance the exposure and release of αPD1 and S1 proteins, promote dendritic cells (DCs) maturation to enhance antigen uptake and presentation, enhance the expressions of CD80 and CD86 on DCs, and prime T‐cell and B‐cell responses, thereby inducing dual antiviral and anticancer immune responses. Furthermore, based on the formation of in situ polydopamine, Pan et al.^[^ ^92^ ^]^ co‐deposited the tumor‐specific antigen ovalbumin (OVA) with dopamine to form antigen‐loaded nanoparticles capable of efficiently adhering to the surface of subcutaneous dendritic cells (Figure 3I). Such chemically‐engineered and in situ polymerization‐mediated antigen presentation undoubtedly enhanced antigen uptake by DCs and promoted lysosomal escape, which in turn enhanced antigen‐specific T‐cell immune responses.

Apart from exogenously binding with functional components, some bacteria themselves could also stimulate the self‐regulation of living body. Cai et al utilized the spontaneous coordination of Ca^2+^ with DOX to obtain DOX‐entrapped CaP/SiO_2_ nanoparticles (DNPs),^[^ ^93^ ^]^ and then modified DNPs with carboxyphenylboronic acid to generate pH‐responsive boronate ester bonds, followed by connection with the probiotic *Bifidobacterium bifidum* (Bi) to obtain the physiochemically‐engineered bacterial (DNPs@Bi). Therein, DOX was triggered to release in the deep tumor and induced tumor cells to undergo immunogenic cell death (ICD). It was worth mentioning that Bi enhanced the expression of tumor cell channel protein Cx43, which in turn facilitated the transfer of tumor‐associated antigens into DCs through Cx43‐dependent gap junctions to induce DCs cross‐presentation. These actions consequently cooperatively activated cytotoxic T lymphocytes, slowed down T cell depletion, and fully activated systemic anti‐tumor immune responses (Figure 3J).

### 2.5 Design Strategies for Combined Physical Therapy

**Figure 4:**
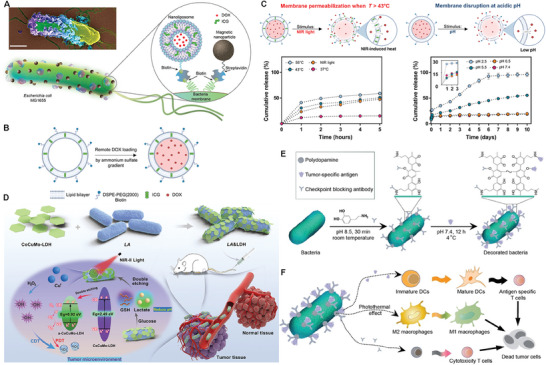
Design strategies for physiochemically‐engineered bacteria‐combined physical therapy. A) Schematic illustration of the bacterial biohybrid microrobots, conjugated with NLs and mNPs. B) Schematic of the NL synthesis. C) Cumulative drug release profile under different conditions. Reproduced with permission.^[^ ^94^ ^]^ Copyright 2022, AAAS. D) Schematic illustration of the preparation of LA&LDH and their in‐situ activation by the TME for tumor‐targeted NIR‐II photodynamic cancer therapy. Reproduced with permission.^[^ ^96^ ^]^ Copyright 2023, John Wiley and Sons. E) Schematic illustration of preparing tumor‐resident living immunotherapeutics by decorating bacteria with triple immune nanoactivators. F) Schematic illustration of decorated bacteria‐mediated reversal of the tumor immunosuppressive microenvironment. Reproduced with permission.^[^ ^97^ ^]^ Copyright 2022, John Wiley and Sons.

Chemically‐engineered bacteria can be integrated with external physical technologies and advanced nanomaterials, wherein the inherent motility of bacteria available for transporting the engineered bacteria to specific environments within the body is preserved. Upon exposure to near infrared stimulation, the cargos can be released to improve the delivery precision and utilization of cargos including drugs and other functional components.^[^ ^51^ ^]^ The surface properties of nanoliposomes permit them to carry hydrophilic, hydrophobic and amphiphilic drugs with larger loading rate. Especially when adjusting the phospholipid membrane properties, the transported drugs can be stimulated to release at temperatures higher than those in normal tissues or in acidic tumor microenvironment when they are exposed to light or other conditions. In a recent study, Metin Sitti et al.^[^ ^94^ ^]^ integrated both nanoliposome (NLs) and magnetic nanoparticles (mNPs) onto Escherichia coli MG1655 via streptavidin and biotin interactions to develop a bacterial biohybrid microrobotic. Under the guidance of magnetic field, iron oxide nanoparticles‐mediated magnetic targeting assisted the bacteria‐based biohybrid microrobot to precisely target tumor (**Figure** **4**A). As well, the encapsulated photothermite indocyanine green (ICG) and DOX that were entrapped within the phospholipid bilayer and the inner aqueous core of nanoliposomes (Figure 4B) could be released under near‐infrared (NIR) light irradiation since the ICG‐mediated photothermal temperature rise and acidic tumor microenvironment can lead to rupture of the nanoliposomes (Figure 4C). This distinctive design combined targeting accumulation, photothermal ablation and chemotherapy in a controlled and precise manner.

Another type of material is 2D layered double hydroxides (LDHs), and their positively charged surface and large surface area determine the applicability of LDH in adsorbing and loading drugs. More significantly, they were sensitive to acidic environment, enabling the acidic tumor microenvironment‐triggered drug release especially after integration with tumor hypoxia zone‐targeted bacteria.^[^ ^95^ ^]^ Typically, Yang and coworkers reported defects‐rich 2D CoCuMo‐LDH nanosheets,^[^ ^96^ ^]^ and they were adsorbed on the surface of *Lactobacillus acidophilus* (LA) under electrostatic interaction to form LA&LDH. The chemically‐engineered probiotic LA&LDH retained the hypoxia targeting property of parent bacteria, which, thereby, not only actively targeted tumors and prolonged the retention time of LA&LDH in tumors, but also metabolized lactic acid to further lower the pH of tumor microenvironment. Inspired by them, 1270 nm laser effectively in‐situ activated the CoCuMo‐LDH nanosheet to produce ^1^O_2_ and achieve efficient PDT (Figure 4D). It is worth noting that the previously‐mentioned PDA featured a high photothermal conversion efficiency, thus dictating that PDA could serve as the trigger. Li et al attached OVA and α‐PD‐1 onto the surface of bacterial EcN via in situ precipitation polymerization of dopamine.^[^ ^97^ ^]^ Assisted by bacterial colonization within the tumor, tumor that experienced near‐infrared irradiation stimulated triple patterns of immune responses, covering DCs maturation, TAMs pro‐repolarization from immunosuppressive M2 macrophages to anti‐tumorigenic M1 phenotype, and cytotoxic T‐cell response potentiation (Figure 4E,F).

In this section, the related strategies and design principles on how to use physiochemical engineering to modify bacterial surface are introduced. In short, based on their exclusive chemical reaction of some specific groups on bacterial surface, stronger covalent bonds are created to promote probiotics to adhere to the host interface. This simple method greatly preserves the inherent biological characteristics of bacteria. Beyond that, physiochemically‐engineered technology combined with advanced nanotechnology to greatly promote the application of bacterial carriers in drug systems in vivo. No matter what self‐assembly, covalent bonding or electrostatic adsorption is used to modify bacterial surfaces, integration with therapeutic drugs brings exogenous new therapeutic effects to bacteria, thus increasing the treatment rates of diseases.

## 3 Genetically‐Engineered Bacteria

Genetic engineering can unite genomics, transcriptomics, proteomics, and metabolomics to design coding gene circuits, enabling engineered bacteria to precisely control gene expression in vivo.^[^ ^41^, ^98^ ^]^ Unlike conventional chemical and biological agents, the sense‐and‐response gene circuits that build genetically engineered bacteria act like an intelligent system dominated by bacterial therapeutic agents, which are able to combine, compute and screen physiological traits through sensing the surroundings (oxygen, pH or lactate) or markers of diseases, thus enabling the autonomous control of bacterial behaviors (localization, growth, invasion, lysis, etc.) in the spatio‐temporal levels.^[^ ^99^ ^]^ In addition, genetic engineering can be attained in the form of gene circuits expressing single target proteins or delivering payloads, and especially boolean‐gated logic circuits can be leveraged to design multi‐modular combinations of gene circuits combining chemotherapeutic drugs, nanomaterials, and physical technologies to further improve sensitivity and stability for therapeutic efficacy.^[^ ^74^, ^100^, ^101^ ^]^ Therefore, rational design of gene circuits in genetically‐engineered bacteria is of great importance because it can control injection location, injection timing, and injection dose of genetically engineered bacterial. In general, gene circuit design strategies have several aims including enhanced specific targeting, host defense evasion, therapeutic playloads transportation, enhanced expansion and quorum‐sensing synchronized lysis circuits and enhanced immune responses (**Table** **2**).^[^ ^102^, ^103^ ^]^

**Table 2:** Representative paradigms of genetically‐engineered bacteria with different design principles.

Design Strategies	Name	Type	References
Genetic circuit design for oncolysis	Three strains of *Escherichia coli*	Cyclical population control	[105]
Genetic circuit design for improving bacterial colonization	Human commensal *Bacteroides thetaiotaomicron*	Phase separation of intrinsically disordered region	[110]
Genetic circuit design for promoting immune regulations	*Escherichia coli* Nissel 1917 (EcN)	Platform of probiotic‐guided CAR‐T cells	[115]
	*Staphylococcus aureus* (S. aureus)	Bio‐engineered CAR‐macrophages	[116]
	The dual genetically‐engineering *Escherichia coli* population	Tumor vaccine treatment	[71]
Genetic circuit design for evading host defense	Salmonella	Intracellular delivery system	[126]
Genetic circuit design for transporting therapeutic payloads	*Clostridium novyi*‐NT (*C. novyi*‐NT)	The group II intron targeting	[127]
Genetically‐engineered microbial chassis fabrication	Native *Escherichia coli* bacteria	Live bacterial therapeutics	[128]
Strategies for fabricating genetically‐engineered bacteriophage	Five bacteriophages, MCoc5c, 8M‐7, 1.2‐3s, KP2‐5‐1 and PKP‐55	Targeting and inhibiting intestinal bacteria	[135]
Modularized multiple‐gene circuit designs for combined treatment actions	*Pseudomonas aeruginosa*	Dynamically programming bacterial lifestyles	[49]
Genetic circuit design for executing biosensing	*Acinetobacter baylyi*	Engineered bacteria themselves as probe	[142]
	*E. coli and Salmonella typhimurium*	Engineered bacteria derivatives as probe	[144]

### 3.1 Genetic Circuit Design for Oncolysis

**Figure 5:**
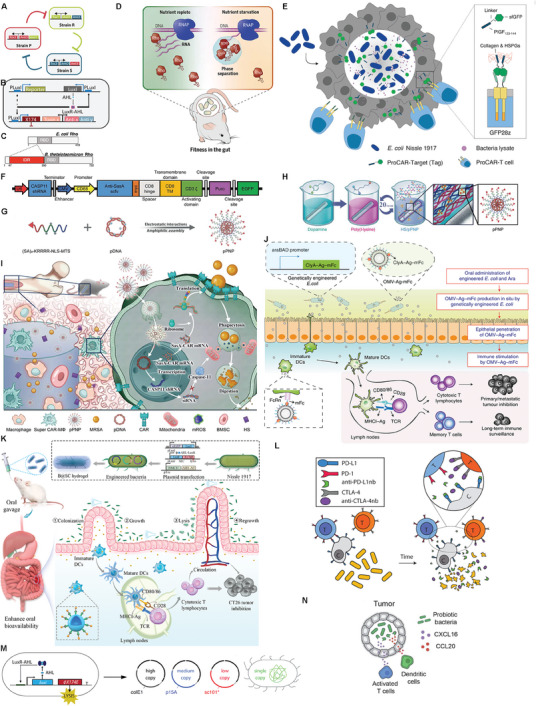
Genetic circuit design for oncolysis and improving bacterial colonization and promoting immune regulations. A) Schematic diagram of sequential strain inhibition of strains. B) Genetic diagram of the quorum‐sensing SLC and TA module. Reproduced with permission.^[^ ^105^ ^]^ Copyright 2019, AAAS. C) Schematic diagram of intrinsically disordered region required in the *B. thetaiotaomicron* Rho protein. D) Phase separation of transcription termination factor Rho in commensal bacterium *B. thetaiotaomicron* governs gene expression and promotes bacterial fitness. Reproduced with permission.^[^ ^110^ ^]^ Copyright 2023, AAAS. E) Schematic demonstrating the ProCAR platform. Reproduced with permission.^[^ ^115^ ^]^ Copyright 2023, AAAS. F) Diagram of anti‐SasA CAR and CASP11 short hairpin RNA structure in plasmid DNA. G) Schematic illustration of the preparation of pDNA‐laden peptide nanoparticle. H) Schematic illustration of the pPNP coating on an implant (Ti‐pPNP). I) Schematic illustration of the locoregional generation of S. aureus–specific super CAR‐MΦs at the bone‐implant interface for preventing periprosthetic joint infection. Reproduced with permission.^[^ ^116^ ^]^ Copyright 2023, AAAS. J) Schematic diagram of genetically engineered bacteria‐derived‐OMV‐based oral tumor vaccine. Reproduced with permission.^[^ ^119^ ^]^ Copyright 2022, Springer Nature. K) Schematic diagram of in situ production and release of nano‐vaccine for tumor immunotherapy by the engineered oral bacterial hydrogel. Reproduced with permission.^[^ ^71^ ^]^ Copyright 2023, Elsevier. L) Schematic showing the mechanism by which engineered bacteria controllably release constitutively produced PD‐L1 and CTLA‐4 blocking nanobodies intratumorally. M) Lysis circuit diagram in which plux drove the transcription of *luxl* and *ϕX174E* genes under a single promoter. Reproduced with permission.^[^ ^120^ ^]^ Copyright 2020, Springer Nature. N) Representative anti‐CD11b‐antibody‐stained sections from the lungs, liver, spleen and bone marrow of mice treated with ANP‐C. Reproduced with permission.^[^ ^121^ ^]^ Copyright 2023, AAAS.

The most typical application of genetic circuit regulation in genetically‐engineered bacteria is to serve as oncolytic bacteria to lyse tumor cells. Similar to oncolytic virus that combined nanoparticles,^[^ ^104^ ^]^ oncolytic bacteria are the most common genetically‐engineered bacteria, which primarily retain their specific replication and expansion in tumor to lyse tumor cells. This process involves the design of quorum‐sensing lysis circuits according to the aims and demands. Inevitably, genetic circuits are subject to the unpredictable effects of mutations. To tackle it, Jeff Hasty's team,^[^ ^105^ ^]^ inspired by the role of ecological complementarity inspired researchers to design a rock‐paper‐scissors (RPS) strategy, and this strategy containing three engineered strains could effectively counteract mutations due to evolutionary selection and stabilize the functioning of genetic circuits (**Figure** **5**A). The researchers constructed specific toxin and antitoxin systems (TA modules) in each strain and introduced a secondary antitoxin to one other strain, in addition to adding the quorum sensing synchronized lysis circuit (SLC) (Figure 5B). This genetic circuit, which is both antagonistic and circular, maintains the population of bacteria in the system and smothers the mutant.

### 3.2 Genetic Circuit Design for Improving Bacterial Colonization

Bacterial colonization is beneficial for regulating intestinal microecology and normalizing intestinal microecology including types and abundance, which is favorable for many gut microbiota‐associated disease treatment since many diseases correlate with the gut microbiota.^[^ ^106^, ^107^, ^108^, ^109^ ^]^ It has been proved that the intrinsically disorder region of Bacteroides polymorphus transcription termination factor Rho was necessary and sufficient for phase separation. (Figure 5C).^[^ ^110^ ^]^ The results showed that the intrinsically disorder region promoted phase separation to isolate Rho from membrane‐free compartments and elevated transcription termination factor activity, thereby controlling bacterial colonization of the gut and altering gene expression to promote bacterial adaptation (Figure 5D). This finding provides new ideas for the use of genetic engineering techniques to manipulate the gut flora to treat diseases.

### 3.3 Genetic Circuit Design for Promoting Immune Regulations

**Figure 6:**
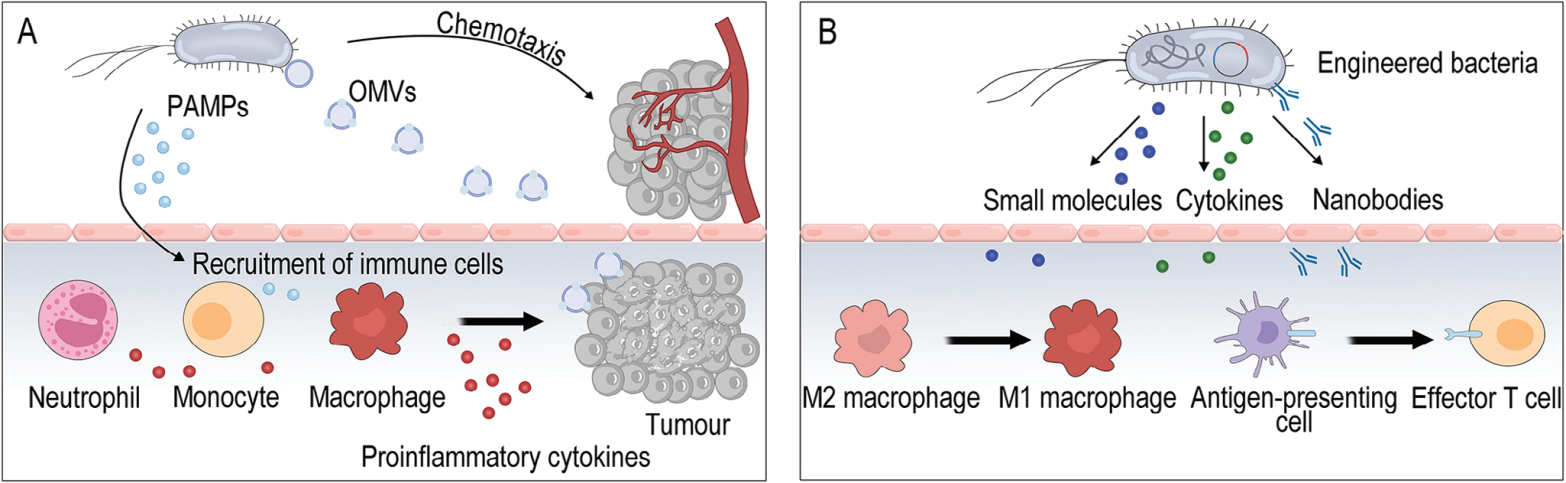
Schematic illustration of bacterial activation of immune system. A) Nanometer‐sized bacterial OMV enrichment in an anoxic tumor environment. B) Engineered bacteria carrying therapeutic cargo targeting immune cells.

Apart from above genetic circuit design for replication and oncolysis, some oncolytic bacteria are genetically engineered to be endowed with regulate immune responses. Naturally, there are abundant PAMPs on the surfaces of bacteria, and these PAMPs can be recognized by Toll‐like receptors (TLRs) or nucleotide binding oligomerization domain‐like receptors on the body's phagocytes. As a result, immune cells can be activated to phagocytose and destroy bacteria, secrete cytokines, and effectively trigger innate immune responses or specific adaptive immunity against tumors.^[^ ^30^, ^111^ ^]^ This process is the direct action mechanism on how genetically‐engineered oncolytic bacteria activate and enhance immune responses. Actually, in most cases, the genetically‐engineered bacteria are armed with more functions or abilities to mitigate immunosuppressive tumor microenvironment, blockade immune escape or evade natural immunity clearance. Bacteria possess a full set of RNA polymerases and can be inserted with transformed gene sequences through recombinant plasmids. Additionally, synthetic biology advances have expedited genetic engineering technologies to alter microorganisms. Inspired by them, genetically‐engineered bacteria can be propelled to produce multiple exogenous proteins and compounds such as antigens, nanobodies, pre‐drug converting enzymes, cytokines, and chemokines in a single strain, thus enabling synergistic therapeutic treatments (**Figure** **6**).^[^ ^112^, ^113^, ^114^ ^]^

#### 3.3.1 Genetic Engineering for CAR‐T Immunotherapy

As a cell therapy, chimeric antigen receptor T‐cell immunotherapy (CAR‐T) has witnessed the prosperity and advances of immunotherapy. Differing from current CAR‐T immunotherapy that focus on in vitro genetically‐engineered T cells and then re‐transfusion in sequence, genetically‐engineered bacteria could in‐situ produce chimeric antigen receptors (CARs) and conjugate them with tumor cells and label tumor cells so as to make them recognize by CAR‐T cells. Tal Danino's innovatively combined probiotic‐targeted TME therapy with CAR‐T immunotherapy to create the ProCAR platform.^[^ ^115^ ^]^ Therein, genetically‐engineered *E.coli Nissle* 1917 (EcN) could infiltrate into the core environment of solid tumor to produce and release CAR targets in‐situ, efficiently labelling tumor cells for recognition and aggregation by guided CAR‐T cells. The CAR target was composed of the heparin‐binding domain of placenta growth factor‐2 (PlGF‐2_123‐144_) and the superfolder green fluorescent protein (GFP). CAR‐T cells were designed to sense and respond to antigenic targets released by genetically‐engineered bacteria, which in turn killed these “Tag” tumor cells in situ (Figure 5E). In addition, the team also designed a CAR target that released an activating mutant (i.e., CXCL16 ^K42A^) of human chemokine to further enhance the recruitment of CAR‐T cells to tumors and improve the therapeutic response.

#### 3.3.2 Genetic Engineering for CAR‐Macrophage Against Bacteria Injection

Besides CAR‐T cells, genetically‐engineered bacteria can also be extended to arm other immune cells in vivo, e.g., macrophages that serve as the innate immune source, and the in vivo bio‐engineered CAR‐macrophages show a general application domain. It has been reported that *Staphylococcus aureus* (*S. aureus*) surface protein A (SasA) that decides periprosthetic joint infection can facilitate bacterial adhesion to host cells and tissues, and help bacteria to achieve immune escape in macrophages, leading to the prolonged infection. To cope with it, a CAR‐macrophage (CAR‐MΦs) based on genetically‐engineered bacteria was developed,^[^ ^116^ ^]^ where genetic engineering technology was harnessed to give birth to functional coatings on bactericidal super CAR‐MΦs in joint prostheses in‐situ. The coating is composed of plasmid DNA, which contains anti‐SasA CAR and caspase‐11 short hairpin RNA (CASP11 shRNA), and heparin sulfate, which contains bone‐enhancing capability. These super CAR‐MΦs can achieve efficient targeting, phagocytosis and elimination of *S. aureus*. (Figure 5F–I).

#### 3.3.3 Tumor Vaccine Treatment

Genetically‐engineered bacteria in tumor vaccines also holds high promise, wherein bacteria acted as highly effective adjuvants and tumor‐targeting delivery vectors to stimulate the immune system to recognize cancer cells and attack them.^[^ ^35^, ^36^ ^]^ In light of the fact that OMVs as therapeutic targets have enlighten more approaches in terms of its action mechanism in regulating gut,^[^ ^80^, ^117^ ^]^ Cheng et al used recombinant DNA technology to fuse tumor antigenic peptides onto the ClyA protein on the surface of OMVs, which in turn enabled the rapid display of multiple different antigenic tumors on the surface of OMVs, developing a genetically‐engineered bacterial‐derived “plug‐and‐play” tumor vaccine platform.^[^ ^118^ ^]^ Furthermore, an oral tumor vaccine (OMV‐Ag‐mFc) was developed to effectively overcome the complex gastrointestinal environment and the intestinal epithelium barrier.^[^ ^119^ ^]^ First, the Fc fragment of mouse immunoglobulin G (IgG; mFc) was fused with tumor antigen (Ag) and further liked to the C‐terminus of protein ClyA (ClyA‐Ag‐mFc), and the recombinant plasmid was transported into bacteria and obtained the genetically‐engineered *E.coli* (Figure 5J). Experimental results showed that the fusion of ClyA with mFc was favorable for the penetration of OMVs secreted by genetically‐engineered *E.coli* across the intestinal epithelial barrier. To prevent long‐term antigenic stimulation from triggering immune tolerance in the body, an arabinose (Ara)‐induced promoter was introduced to control the expression of fusion protein. Oral genetically‐engineered bacteria under the control of Ara generated OMVs loaded with tumor antigens in situ in the intestine, and the OMVs were recognized and absorbed by DCs in the lamina propria through the interaction between mFc and neonatal Fc receptor (FcRn), which stimulated DCs maturation and draining to the lymph nodes and activated antigen‐specific T cells to fight against tumor cells. In both lung metastasis melanoma and subcutaneous colon tumors, OMV‐Ag‐mFc significantly inhibited tumor growth. This work pointed out a feasible avenue to develop oral tumor vaccine.

In addition, dual gene circuit designs are encouraged to fabricate bacteria vaccines featuring stronger immune activation. As a paradigm, chimeric gene was integrated translationally into Escherichia coli; and the antigenic peptide AH1‐A5 (one component of envelope protein MuLV GP70,) was also fused to the C‐terminal H subunit of bacterial micro‐component (BMC‐H). The dual genetically‐engineering Escherichia coli population was programmed by synchronous lysis circuit (SLC) to realize routine and controlled in‐situ lysis and accumulative release of nanovaccines (Figure 5K).^[^ ^71^ ^]^ The subcutaneous tumor model further confirmed that the engineered bacteria could produce and release nanovaccines in vivo, effectively activate mesenteric lymph nodes and spleen immune cells, trigger systemic immunity, and thus inhibit the growth of subcutaneous tumor. This kind of engineering bacteria which used synthetic biology to construct group susceptibility cleavage and release nano‐vaccine greatly improved the safety and therapeutic effect of oral nanovaccines.

#### 3.3.4 Genetic Engineering for Immune Checkpoint Blockade Therapy

It has been recorded that genetically‐engineered bacteria could not only control the rate of bacterial production within the tumor and improve therapeutic safety, but also exerted the abscopal effect against metastatic lesions. With probiotic *E.coli* Nissle 1917 (EcN) serving as a therapeutic vector,^[^ ^120^ ^]^ blocking nanobody sequences of programmed cell death protein‐ligand 1 (PD‐L1) and cytotoxic T‐lymphocyte‐associated protein‐4 (CTLA‐4) were cloned into a high‐copy plasmid, followed by the transfection into the EcN‐lux genome for expressing anti‐PD‐L1 and anti‐CDLA‐4 nanobodies (Figure 5L,M). The researchers used computational modelling and lysis circuit dynamics experiments to select the genetic circuit parameters under which the release of therapeutic drug in a synchronized lysing integrated circuit (SLIC) system was optimized. In a mouse model of lymphoma, it was verified that the injection of genetically engineered bacterium SLIC‐2 (co‐expressing anti‐PD‐L1nb and anti‐CTLA‐4 nb in equal amounts) resulted in better therapeutic efficacy than SLIC:PD‐L1nb alone or SLIC:CTLA‐4 nb alone.^[^ ^112^ ^]^ Contributed by them, an increase in activated T‐cells (CD8^+^IFNγ^+^TNFα^+^) and a decrease in regulatory T‐cells (CD4^+^ FOXP3^+^) were accessible. Briefly, the SLIC system provided a successful paradigm associated with the combination of an optimized lysis mechanism with enhanced cancer immunotherapy.

#### 3.3.5 Genetic Engineering for Cytokine Therapy

Anti‐tumor cytokine secretion by genetically‐engineered bacteria provide another choice to enhance immunotherapy via supplementing the inadequacy of autologous secretion. In a recent report, Thomas and co‐workers constructed genetically‐engineered *E.coli* (eSLC‐hCXCL16^K42A^ and eSLC‐CCL20) to trigger the synchronous lysis circuit (SLC) after colonization in tumors,^[^ ^121^ ^]^ and the engineered bacteria expressed the activating mutants of human chemokines CXCL16 (hCXCL16^K42A^) and CCL20. CXCL16 promoted cytotoxic T cell chemotaxis and thus attracted immune cells into the tumor microenvironment. In addition, it also exerted a synergistic interaction with CCL20 to enhance the recruitment of type‐1 conventional DCs, strengthened anti‐tumor immune responses, and promoted tumor regression (Figure 5N). This work also offered a successful paradigm that integrated tumor lysis with cancer immunotherapy in genetically‐engineered bacteria.

### 3.4 Genetic Circuit Design for Evading Host Defense

**Figure 7:**
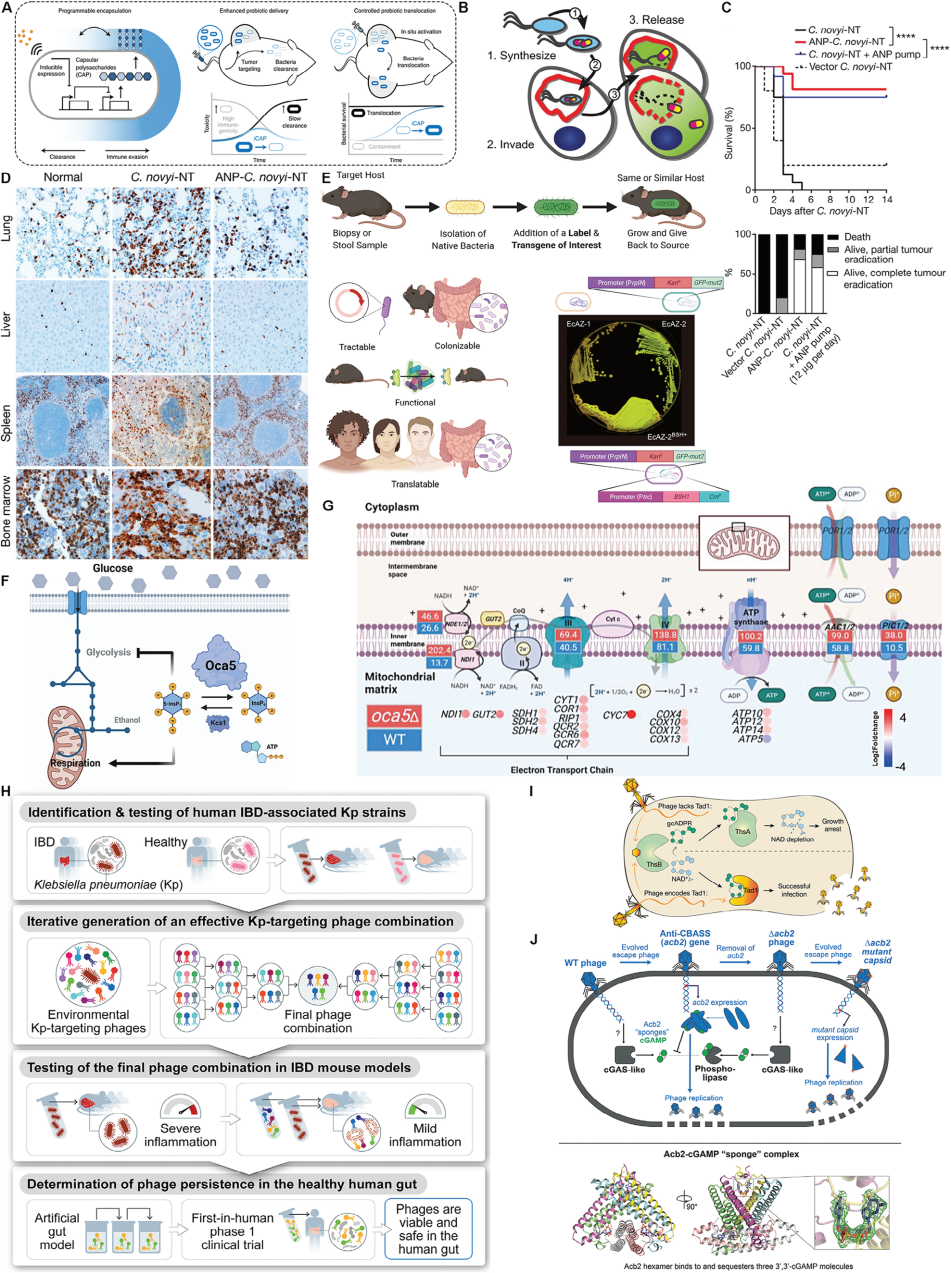
Genetic circuit design for evading host defense and transporting therapeutic payloads, and strategies for fabricating genetically‐engineered microbial chassis and bacteriophage. A) Programmable CAP system for control over bacterial encapsulation and in vivo delivery profiles. Reproduced with permission.^[^ ^122^ ^]^ Copyright 2022, Springer Nature. B) Schematic diagram of designing a bacterial vector by genetic engineering. Reproduced with permission.^[^ ^126^ ^]^ Copyright 2021, Springer Nature. C) Kaplan–Meier curve (top panel) and therapeutic response (bottom panel) of ANP‐C. D) Representative anti‐CD11b‐antibody‐stained sections from the lungs, liver, spleen and bone marrow of mice treated with ANP‐C. Reproduced with permission.^[^ ^127^ ^]^ Copyright 2018, Springer Nature. E) Experimental strategy of engineered native bacteria. Reproduced with permission.^[^ ^128^ ^]^ Copyright 2022, Cell Press. F) A hybrid‐glycolysis yeast that disrupts the Embden‐Meyerhof‐Parnas glycolysis pathway and introduced components of the phosphoketolase pathway. G) Schematic diagram of fluxes and gene expression for the electron transport chain and ATP synthase. Reproduced with permission.^[^ ^130^ ^]^ Copyright 2023, Cell Press. H) Schematic diagram of phage consortium targeting inhibition of human IBD‐related intestinal microflora symbionts in the treatment of intestinal inflammation. Reproduced with permission.^[^ ^135^ ^]^ Copyright 2022, Cell Press. I) Mechanism model diagram of Tad1. Reproduced with permission.^[^ ^136^ ^]^ Copyright 2022, Springer Nature. J) Bacteriophages antagonize cGAS‐like bacterial immunity by sequestering immune signaling molecules and acquiring capsid gene mutations. Reproduced with permission.^[^ ^137^ ^]^ Copyright 2023, Cell Press.

Bacterial surface capsular polysaccharide (CAP) can cover the extracellular membrane, which not only facilitates microbial resistance to acidic environment, but also evades host immune surveillance, interferes with phagocytosis by macrophages, hinders clearance by host complement, and promotes bacterial survival and colonization. Based on CAP synthesis, Harimoto et al developed genetically‐encoded microbial encapsulation system based on EcN, namely the inducible CAP (iCAP) system (**Figure** **7**A).^[^ ^122^ ^]^ In detail, small RNA knockdown screen for identifying key CAP genes capable of altering response to antibacterial factors was carried out, and the kfiC gene that was a glyco‐transferase encoding N‐acetyl‐α‐D‐glucosamine (GlcNAc) to regulate the density of CAP was screened out. It was reported that ΔkfiC correlate with cell protection against antibiotics and extreme acidic environments, and kfiC deletion resulted in the abrogation of CAP synthesis. In subsequent experiments, inducible synthetic gene circuits were constructed in *EcN*, in which the kfiC gene was cloned under the control of lac promoter, and the small‐molecule inducer isopropyl‐β‐D‐thiogalactopyranoside activated the lac promoter to induce an increased CAP synthesis. In short, such iCAP system could be modulated by external inducers through dynamically regulating the expression of surface CAP, and transient CAP allowed injected bacteria to temporarily evade immune attack, and thus increased bacterial accumulation and oncolysis for primary and distant tumors.

Salmonella is a common gram‐negative bacillus (subordinate to the *Enterobacteriaceae* family), and known for the intracellular bacteria.^[^ ^123^ ^]^ Salmonella secretes effector proteins that can be released intracellularly via the type III secretion system (T3SS) to modulate host cell signaling and physiological activities.^[^ ^124^ ^]^ T3SS not only controls Salmonella entry into epithelial cells, but also regulates intracellular formation of *Salmonella*‐containing vacuole (SCV) that provides the necessary environment for the survival and proliferation of Salmonella.^[^ ^125^ ^]^ The special physiology of *Salmonella* can be leveraged to exploit its mechanisms of host invasion and defense, and especially the combination with bioengineering techniques provides new routes to deliver drugs and treat diseases. Vishnu Raman's team constructed the intracellular delivering *Salmonella* platform that contained three gene circuits associated with the abilities to precisely control the synthesis of drug proteins, and efficiently invade tumor cells, and release the proteins (Figure 7B).^[^ ^126^ ^]^ Researchers activated the regulator *flhDC* expression through the constructed gene circuit (*PBAD‐flhDC*), and further regulated the production of flagella and T3SS‐1 from the bacteria, evading the host's defense mechanisms. It was obtained that the engineered bacteria invaded 84% of cells and transported therapeutic proteins into cancer cells over 500 times. When the bacteria invaded the cell, the genetic circuit *PsseJ‐LysE* came into play. It was worth noting that the *PsseJ* promoter was first activated only in SCVs, and the proteins released by bacterial lysis and death were deposited intracellularly to interact with their targets before their entry into the cytoplasm. In mouse models of hepatocellular carcinoma and triple‐negative breast cancer, constitutively active caspase‐3 blocks‐producing *Salmonella* induced a reduction in tumor growth and blocked the growth of metastatic foci in the lungs, increasing the survival rate of tumor‐bearing mice.

### 3.5 Genetic Circuit Design for Transporting Therapeutic Payloads

The use of genetically‐engineered bacteria for transporting protein drugs into the cell is undoubtedly a way to improve the efficacy and broaden the therapeutic avenues for solid tumors. The naerobic spore‐forming bacterial strain *Clostridium novyi*‐NT (*C. novyi*‐NT) is often used as a therapeutic agent because of its ability to penetrate deep tissues to colonize and cause damages in the hypoxic tumor environment.^[^ ^22^ ^]^ However, high doses during treatment are demanded, which inevitably triggers sepsis or cytokine release syndrome (CRS) and organ failure. To tackle it, Zhou et al. engineered atrial natriuretic peptide (ANP)‐secreted *C. novyi*‐NT (ANP‐*C. novyi*‐NT) by using the group II intron targeting to stably integrate ANP with a signal peptide into C. novyi‐NT genome and obtain.^[^ ^127^ ^]^ The tumor‐bearing mice treated with ANP‐*C. novyi*‐NT showed significant tumor regression and higher survival rate compared with those before genetic modification. Importantly, tumor‐bearing mice expressed bits of pro‐inflammatory factors in their peripheral blood and decreased immune cell infiltration associated with cytokine storms (Figure 7C and D). This study provided a valuable clinical strategy for the treatment of inflammatory injury and cytokine release syndrome induced by biotherapeutics such as CAR‐T, T‐cell‐targeted antibodies, and genetically‐engineered tumor‐loving bacteria.

### 3.6 Genetically‐Engineered Microbial Chassis Fabrication

A well‐designed microbial chassis can specifically enable persistent biofactories, which aroused increasing interests since it could disrupt the abundance and type of host gut microbiome to interfering disease progression. Amir's team used natural *E.coli* isolated from mouse fecal cultures as the microbial chassis EcAZ‐1,^[^ ^128^ ^]^ and they first transduced them with phage for GFP, and subsequently with bile salt hydrolase (BSH) (EcAZ‐2^BSH+^) and cytokine IL‐10 (EcAZ‐2^IL10+^). The engineered bacteria that could express functional genes were re‐transplanted into the host (Figure 7E). EcAZ‐2 could stably and permanently colonize the conventionally raised (CR) host gut after only a single gavage treatment. Noticeably, this genetically‐engineered bacterium caused changes in metabolites in vivo without affecting the overall composition of host gut microbiome, improving the treatment outcome of chronic diseases such as type 2 diabetes. Similarly, attenuated version of *M. pneumoniae* M129 strain as a bacterial chassis was also available for the treatment and prevention of infectious lung diseases caused by *Pseudomonas aeruginosa*.^[^ ^129^ ^]^

The primary way that allows most microorganisms to obtain energy is the glycolytic pathway where glucose is degraded to produce ATP and thus supply energy for life activities. It has been reported that 5‐diphosphoinositol 1,2,3,4,6‐pentakisphosphate (5‐InsP_7_) senses cellular ATP levels and regulates central carbon metabolism macroscopically, and the reciprocal regulation of respiratory and metabolic fluxes is also an important area of research in cancer therapy. In a recent study, Qin et al identified Oca5 as an inositol pyrophosphatase of *S. cerevisiae* that could catalyze the conversion of the “energy receptor” 5‐InsP_7_ into inositol hexakisphosphate (InsP_6_).^[^ ^130^ ^]^ InsP_6_ in turn regulated the pyrophosphorylation of the transcription factor Gcr1 to control glycolysis and realize respiration regulation through the Mig1/Hap4 signaling pathway (Figure 7F). In their work, the researchers first constructed a hybrid‐glycolysis yeast (QL5) that linked the Embden‐Meyerhof‐Parnas (EMP) pathway to the phosphoketolase (PK) pathway via the oxidized PP (oxPP) pathway. Such a design contributes to the understanding of the relationship between eukaryotic glycolysis and energy metabolism in eukaryotes. Further, the knockdown of OCA5 in QL5 and *Saccharomyces cerevisiae* WT brought about glucose consumption limitation and elevated the energy metabolism efficiency, resulting in the increase in the ratio of 5‐InsP_7_ to InsP_6_ (Figure 7G). This finding is an extension of inositol phosphate signaling pathway and also has important implications for hybrid glycolytic yeast as a novel chassis cell factory.

### 3.7 Strategies for Fabricating Genetically‐Engineered Bacteriophage

Genetically‐engineered bacteriophages can enter bacteria with the help of different receptors and induce bacteria lysis, which is mainly used for treatment against infection caused by drug‐resistant bacteria.^[^ ^131^, ^132^, ^133^ ^]^ Based on the principle of high‐throughput sequencing, accurate genome editing and bacteriophage design guided by artificial intelligence,^[^ ^134^ ^]^ Elinav et al.^[^ ^135^ ^]^ screened five bacteriophages, MCoc5c, 8M‐7, 1.2‐3s, KP2‐5‐1 and PKP‐55, and then combined them. This treatment strategy could accurately target and inhibit intestinal bacteria related to IBD (Figure 7H).

In the bacterial antiphage system, Thoeris, the Toll/interleukin‐1 receptor domains, are one key component of bacterial in recognizing pathogen invasion and triggering immune response. Sorek's team found that the Thoeris anti‐defence 1 (Tad1) protein in bacteriophage could specifically bind and sequester the immune signalling molecule 1′'−3′ gcADPR,^[^ ^136^ ^]^ thus decoupling bacteriophage sensing from immune effector activation and rendering Thoeris inactive (Figure 7I) since Tad1 is the regulation gene of Thoeris‐inhibited SBSphiJ7 (a Myoviridae phage) phenotype. This mechanism of immune evasive action demonstrated a novel mode of host inhibition by pathogens, which pointed out a new direction for engineering bacteriophage. In addition, similar to CRISPR‐related systems, cyclic‐oligonucleotide‐based antiphage signaling system (CBASS) is another immune defense system of bacteria. At present, the molecular mechanism of bacteriophage inhibition and escape from the immune system of CBASS has been revealed. It has been found that bacteriophage can effectively destroy the immune function of CBASS system by expressing anti‐CBASS protein, Acb2, which adsorb and isolate 3 ‘, 3′‐cGAMP produced by bacteria (Figure 7J).^[^ ^137^ ^]^ Additionally, Acb2 deletion will cause CBASS to block bacteriophage replication and lysogen induction, but a few of bacteriophages will escape the immune effect of CBASS through the mutation of major capsid genes.

### 3.8 Modularized Multiple‐Gene Circuit Designs for Combined Treatment Actions

**Figure 8:**
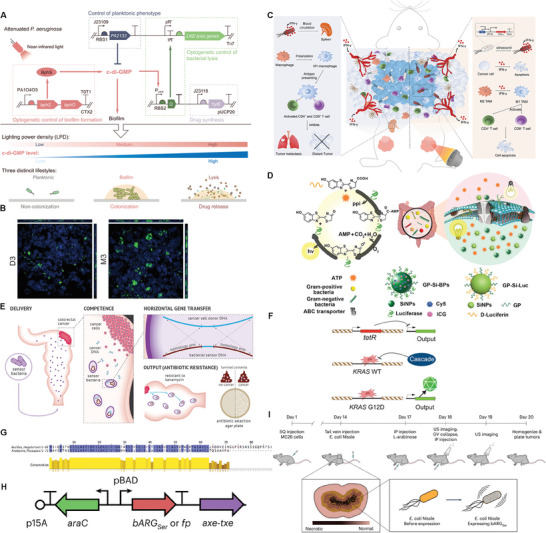
Modularized multiple‐gene circuit designs for combined treatment actions. A) Schematic illustration of genetic circuit design for programming bacterial lifestyles. B) Representative confocal microscopy image of frozen tumor sections taken from mice in group D3 and M3. Reproduced with permission.^[^ ^49^ ^]^ Copyright 2023, Science China Press. C) Schematic diagram of ultrasound‐responsive bacteria in controlling IFN‐γ expression by focused ultrasound and their mechanisms for cancer immunotherapy. Reproduced with permission.^[^ ^140^ ^]^ Copyright 2022, Springer Nature. D) Schematic design of ABC sugar transporter. Reproduced with permission.^[^ ^141^ ^]^ Copyright 2023, Springer Nature. E) Schematic diagram of engineered bacteria for detecting tumor DNA. F) Schematic diagram of intrinsically disordered region required in the B. thetaiotaomicron Rho protein. Reproduced with permission.^[^ ^142^ ^]^ Copyright 2023, AAAS. G) Schematic diagram of sequence homology of GvpA/B. Reproduced with permission.^[^ ^144^ ^]^ Copyright 2018, Springer Nature. H) Diagram of the construct from a with Axe‐Txe47 added, creating pBAD‐bARGSer‐AxeTxe, to enable plasmid maintenance in the absence of antibiotics. I) Diagram of the in vivo protocol for assessing in situ bARGSer expression in tumors. Reproduced with permission.^[^ ^145^ ^]^ Copyright 2023, Springer Nature.

Multifunctional genetically‐engineered bacteria can realize the modular design of gene circuits by individualizing the main functions of each module. This design can realize drug synthesis and remote‐controlled release after combining physical triggers such as light and heat. Modularized genetically‐engineered bacteria are highly automated pharmaceuticals that work long‐term at colonization sites in the body, which are expected to kill malignant cells directly or regulate the immune system indirectly, and coincidently improve patient compliance.^[^ ^138^ ^]^ Herein, an engineering strain that could program bacterial lifestyle (plankton, biofilm or lysis) by hierarchically regulating near‐infrared light intensity was developed.^[^ ^49^ ^]^ The genetic circuit of such engineered strain was composed of a NIR sensor module and four genetic programming modules, and it mainly affected the intracellular concentration of the second messenger cyclic di‐guanosine monophosphate (c‐di‐GMP) by controlling the amount of activated BphS proteins with variable light power density (LPD), thus controlling the bacterial lifestyle (**Figure** **8**A). Notably, the molecular mechanism on how LPDs affected the different lifestyles of bacteria was jointly uncovered by monitoring the ribosome‐binding site upstream of PA2133 (RBS1) and the ribosome‐binding site upstream of anti‐termination protein Q (RBS2). In addition, after injecting genetically‐engineered bacterial (H017) in the subcutaneous A549 tumor model, H017 failed to colonize tumor tissues in the absence of NIR illumination, while biofilms were generated in the tumors after 3 days (M3) of NIR illumination at medium‐LPD (1.2 mW cm^−2^) (Figure 8B). In contrast, the colonies were fully lysed under NIR irradiation at high‐LPD, suggesting that NIR sensitivity and lysis ability remain unchanged in vivo. The programmed and precise control of bacterial global phenotype and properties in tumor including bacterial adhesion, intratumoral colonization and explanatory drugs for bacterial cleavage, was realized by adjusting the lysis circuit in photoactivated genetically‐engineering bacteria, showing remarkable advantages in the long‐term treatment of tumor.

Sense‐response gene circuits can control gene switches based on temperature‐driven control, and especially focused ultrasound capable of penetrating tissues to cause transient hyperthermia at a specific site enables remote the control of gene expression and avoids the non‐specific colonization of bacteria delivery vectors in other places.^[^ ^139^ ^]^ The leftward (PL) and rightward (PR) phage lambda promoters were temperature‐sensitive elements, and they inactivated the TcI repressor when the temperature increased to 42–45 °C, triggering transcription under the pR‐pL promoter genes. Based on them, Chen et al inserted a clone expressing the cytokine interferon‐γ (IFN‐γ) (or the mCherry gene) under pR‐pL tandem promoter,^[^ ^140^ ^]^ and the recombinant plasmid was transformed into *E.coli (*MG1655) to successfully yield ultrasound‐responsive bacteria (Figure 8C). Under focused ultrasound irradiation and heating, a precise increase in deep tissue temperature triggered IFN‐γ production and secretion, which in turn activated Janus kinase 1 (JAK1), and up‐regulated the signal transducer and activator of transcription 1 (STAT1) signaling pathways. These actions not only promoted apoptosis of cancer cells, but also induced macrophage polarization, which led to the activation of CD4^+^ and CD8^+^ T cells.

### 3.9 Genetic Circuit Design for Executing Biosensing

#### 3.9.1 Engineered Bacteria Themselves as Probe

Genetic circuit design can be also expanded to other application according to their various demands. There are significant advances in the application of engineered bacteria for diagnostic imaging. Typically, specifically genetically‐engineered bacteria that highly expressed ATP‐binding cassette (ABC) sugar transporters were obtained. These ABC‐expressed bacteria could engulf one composite nanoprobe consisting of Cyanine 5(Cy5), ICG, and glucose polymer (GP)‐ and D luciferase‐modified silicon nanoparticles (SiNPs) (GP‐Si‐BPs), which allowed ICG and SiNPs (GP‐Si‐Luc) to be internalized into bacterial cells and image deep tissues since ICG and SiNPs could emit near‐infrared fluorescence (Figure 8D).^[^ ^141^ ^]^ Beyond in vivo imaging, genetically‐engineered bacteria could be designed to detect free DNA in vivo from donors. Based on the natural sensory state of *Acinetobacter baylyi (A. baylyi)*, Cooper et al designed a targeting, CRISPR‐discriminated horizontal gene transfer (CATCH) cellular detection strategy for detecting free‐floating DNA sequences at the genome level through comparing them with preset cancer sequences.^[^ ^142^ ^]^ Therein, they programmed *Acinetobacter baylyi* by using CRISPR system to insert tetR suppressor gene between KRAS homologous arms of biosensor and place the output gene controlled by P_LtetO‐1 promoter at the second site, successfully obtaining in vivo biosensor for finding mutation form of KRAS gene (Figure 8E,F). Based on the fact that the target sequence and coding output genes were modularly designable, such genetic circuit for targeting tumor DNA detection provided more sensitive diagnostic methods in clinical medicine.

#### 3.9.2 Engineered Bacteria Derivatives as Probe

Gas vesicles (GVs) are protein nanostructures found primarily in planktonic organisms or archaea, and as bacteria one derivative, they can regulate buoyancy by allowing free passage of gases within them,^[^ ^143^ ^]^ which are identified as a promising target for acoustic response design.

Based on the unique structure and gene editing proteins of GVs, Shapiro's team first synthesized the first generation acoustic reporter gene (ARG) by combining GvpA gene and GvpC gene from *A. flos‐aquae* with GvpR‐GvpU genes from *B. megaterium*.^[^ ^144^ ^]^ After transfecting ARG1 in *E.coli* and *Salmonella typhimurium*, gas vesicles that could be recognized by ultrasound visualization were successfully expressed (Figure 8G). Furthermore, their group screened and optimized the genome of GVs to construct two new ARGs: bARG_Ser_ and mARG_Ana_, to address the previous difficulty in distinguishing GVs from background tissues due to their linear scattering.^[^ ^145^ ^]^ Especially GVs destruction‐arised signal disappearance enabled the background‐free ultrasound visualization through subtracting the post‐destruction ultrasound signal. In addition, the gene cluster bARG_Ser_ conjugated to the pBAD promoter and the toxin‐antitoxin stabilization cassette Axe‐Txe47 (pBAD‐bARG_Ser_‐AxeTxe) were simultaneously transfected into *E.coli* (Figure 8H), and the genetically‐engineered bacterial not only enabled tracking of therapeutic bacteria and targeted colonization in tumors, but also enabled ultrasound imaging of prolonged and stable expression in the presence of L‐arabinose inducers (Figure 8I). Apart from imaging, Yang et al transformed the genes encoding GVs (GvpA and GvpC and GvpR‐GvpU) into the *E.coli* BL21 (GVs@*E.coli*) to manipulate the movement direction of GVs@*E.coli* in the vasculature of live mice using acoustic tweezers,^[^ ^146^ ^]^ and increase the aggregation efficiency in tumors for effectively inhibiting tumor growth.

## 4 Genetically and Physiochemically‐Combined Engineered Bacteria

**Figure 9:**
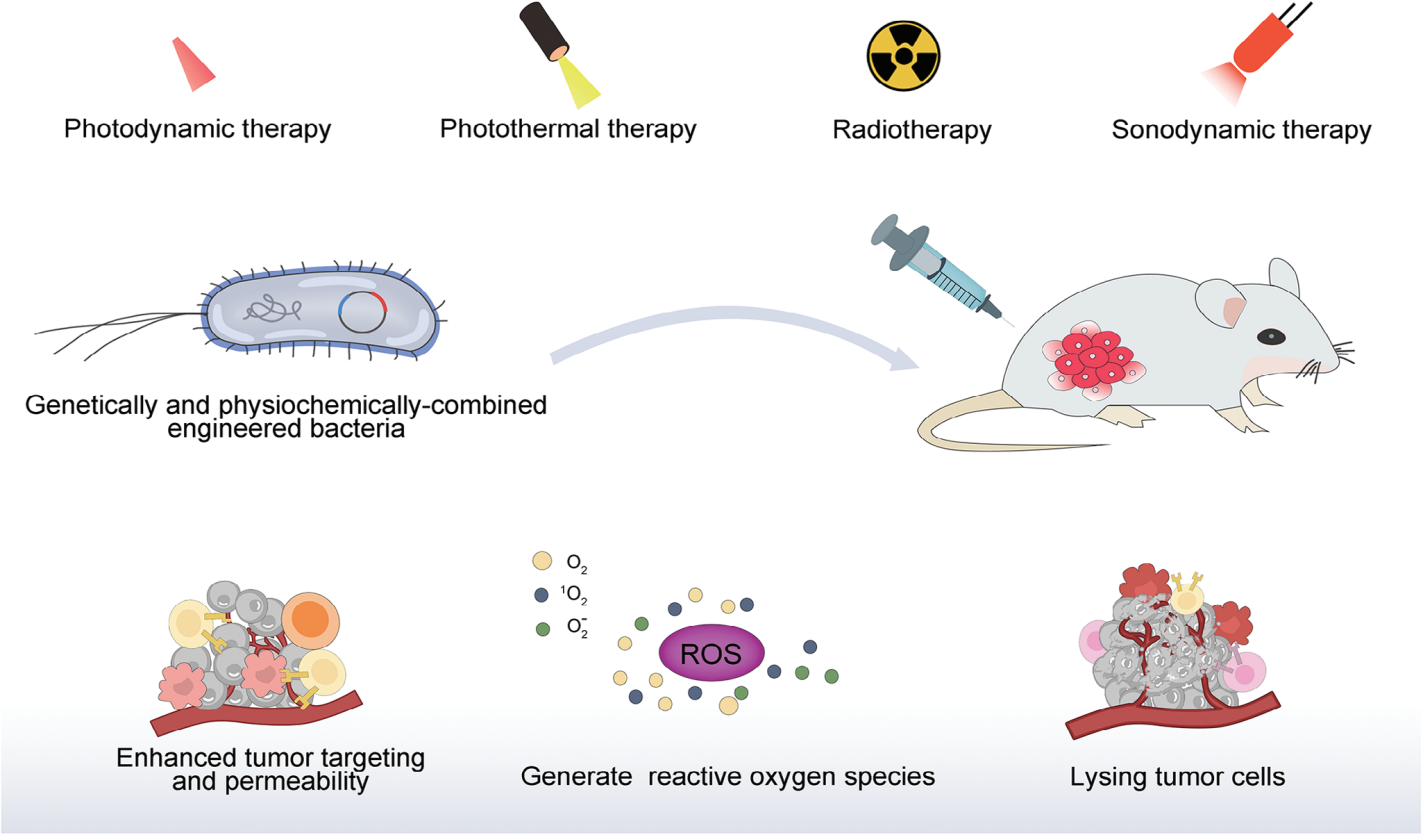
Schematic illustration of physiochemically‐ or/and genetically‐engineered bacteria combined with physical therapy. Genetically and physiochemically‐combined engineered bacteria can be combined with physical therapies such as PDT, PTT, SDT and RT to enhance tumor targeting and permeability, and produce cytotoxic reactive oxygen species, thus destroying tumor cells.

Aiming at the complicated microenvironment and pathogenesis, supplementary mechanisms often compromise single therapy, which, thereby, determine that combined therapy is highly desirable. Engineered bacteria are not the exception, and various combined therapeutic protocols have been developed,^[^ ^6^, ^19^, ^147^ ^]^ which is not only limited to active tropism‐rooted drug delivery‐based combined therapies. As a result, multiple physiochemically‐ or/and genetically‐engineered bacteria were designed and constructed to realize the various certain demands (**Figure** **9**).

### 4.1 ROS‐Combined Therapy

ROS is a double‐edged sword.^[^ ^148^ ^]^ In normal tissues, reducing radiation‐induced excessive ROS can effectively evade the destruction of redox balance and attenuate the damages brought by biomolecules. On the contrary, augmenting ROS level provides reference for oncolytic treatment. Sun et al. developed the micro‐to‐nano oncolytic microbial therapeutics (Ecp@TAPP NPs‐PMAN) that utilized the biological characteristics of bacterial OMV. Genetically‐engineered Escherichia coli that could highly express pyranose oxidase was chemically decorated with 4‐aminophenyl‐dmannopyranoside (MAN)‐capped amide polyethylene glycol (PEG) chains (NH_2_‐PEG‐MAN, PMAN) by bio‐condensation reaction. After the intratumoral injection of EcP@TAPP NPs‐PMAN, a large amount of hydrogen peroxide was produced to kill tumor cells and release tumor antigen in the presence of laser irradiation. Furthermore, the released OMV remodeled the immune conditions of tumor‐draining lymph nodes for effective oncolytic microbial therapy.^[^ ^149^ ^]^

In the course of bacteria engineering, selecting appropriate bacteria is the premise because different lesion site and varied demands often decide the success or failure of the eventual engineered bacteria. In recent years, lactic acid bacteria (LAB) as mucosal vectors have attracted wide attention in intestinal tract disease. Plasmids were introduced by genetic tools into LAB to stably express foreign proteins, and then caused strong immune responses after colonization in intestinal tract in vivo. After genetical engineering that co‐expressed cytokines, the therapeutic and preventive molecular delivery of LAB vaccines was further optimized to enhance its immunogenicity.^[^ ^150^ ^]^

### 4.2 Thermal Ablation‐Combined Therapy

**Figure 10:**
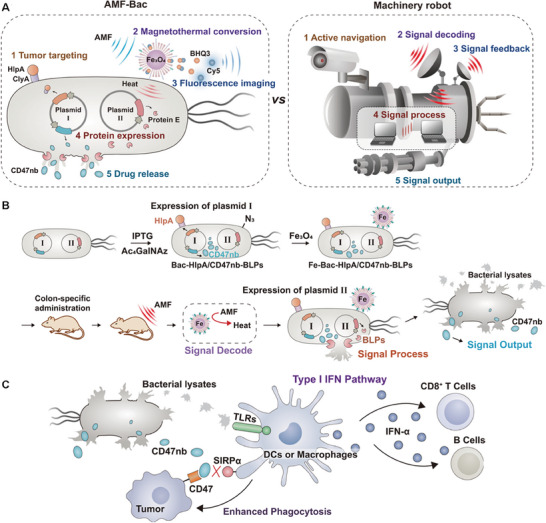
Genetic circuit design for executing biosensing. A) An illustration of AMF‐Bac, comprising five modules. active navigation, signal decoding, signal feedback, signal process, and signal output. B) An illustration of the assembly process and working principle of AMF‐Bac. C) Schematic illustration of the type I IFN pathway and adaptive immunity activated by AMF‐Bac. Reproduced with permission.^[^ ^154^ ^]^ Copyright 2025, Springer Nature.

Thermal ablation is common therapeutic method, which have been verified to be combined with many therapeutic protocols and biomaterials.^[^ ^151^, ^152^, ^153^ ^]^ Based on the magnetocaloric effect of magnetic nanomaterials, the time‐space manipulation of bacterial gene expression and drug release behavior in vivo by alternating magnetic field (AMF) can be realized. Ma et al designed AMF‐manipulated tumor‐homing bacteria (AMF‐Bac) that were physiochemically‐ and genetically‐engineered to encompass five main functional modules (**Figure** **10**A).^[^ ^154^ ^]^ Therein, genetically‐engineered *E.coli* BL21 that could express fusion proteins (ClyA‐HlpA) and enhanced GFP under the inducible promoter (isopropyl‐β‐d‐thiogalactoside) were constructed to specifically target tumor cells with highly‐expressed heparan sulfate proteoglycans. Furthermore, paramagnetic Fe_3_O_4_@lipid nanocomposites (DBCO‐Fe_3_O_4_) with dibenzocyclooctyne (‐DBCO) molecules on the surface were then used to connect the engineered bacteria via the click chemistry reaction between ‐DBCO and ‐N_3_ moieties. Notably, conjugated Fe_3_O_4_ nanoparticles could respond to AMF signals and convert them to heat, and the magneto‐thermal signals stimulated low temperature‐sensitive lipid to execute the “signal feedback” module to simultaneously release the fluorescent molecule Cy5 and the quenching chaperone molecule BHQ3 for fluorescence imaging. In addition, under the heat‐sensitive promoter, the engineered bacteria received magnetic heat signals to express bacterial lysing proteins and thus released the antitumor protein CD47nb into tumor tissues (Figure 10B). This study not only significantly increased the targeting accumulation of bacteria in tumors by controlling the movement direction of engineered bacteria with a constant magnetic field, but also effectively enhanced the antigen presenting ability of antigen‐presenting cells (APCs) and activated the type I interferon pathway of APCs by manipulating the release of CD47nb from AMF‐Bac with the help of AMF (Figure 10C).

## 5 Summary and Future Outlook

Currently, microbes have been validated to play multifaceted roles, including detrimental aspects like inflammation aggravation, tumor development and therapeutic effect abrogation, and preferable merits associated with immunity activation, gut homeostasis balance and cancer progression inhibition.^[^ ^155^, ^156^, ^157^ ^]^ Fortunately, the physiochemically‐ and genetically‐engineered bacteria used in therapeutic disease strategies are constantly evolving to defend against unfavorable environments to increase the efficiency of transporting loads, to regulate gut homeostasis and remodel the tumor microenvironment, and to stimulate immune cells to fight tumors. In this review, we specifically and comprehensively expounded the physiochemically‐ and genetically‐engineered bacteria, respectively, and classified and highlighted the engineering rationales, discussed their underlying mechanisms and design principles aiming at different demands.

It has been gradually accepted that physiochemical or genetical engineering has imparted bacteria with more functions, and expanded the application domain, which has aroused increasing interests. They not only improve the resistance of bacteria to harsh environment, enhance targeted positioning, but also make it easier to realize scale production and application. However, there are many concerns and unresolved challenges that need to be addressed, e.g., innate immune response activation and cytokine storm inevitably removed bacteria and discourage the functions of engineered bacteria.

1. The combination of physiochemically‐engineered bacteria enables the precise modulation of engineered bacteria, thereby expanding their functional diversity. This interdisciplinary combination not only promotes the innovation and development of nanobiotechnology, but also plays a pivotal role in developing the safety, efficiency and environmental sustainability of future bioactive materials.^[^ ^158^, ^159^, ^160^ ^]^ For example, the probiotic Bacillus subtilis could reduce the potential liver and kidney damages of African catfish induced by thiamethoxam; and Lactobacillus plantarum L‐137 and/or β‐glucan could significantly improve the feeding efficiency and health status of Nile tilapia. These findings provide important scientific basis for designing and developing novel controlled release systems of biological agents.^[^ ^161^, ^162^ ^]^ The design concept of green biomaterials highlights non‐toxicity and natural degradability to reduce potential damages to normal tissues. Apart from it, more attention is also paid to biocompatibility, aiming to minimize long‐term effects on the host and create a harmonious coexistence microenvironment between biomaterials and host.^[^ ^163^ ^]^
2. It is urgent to solve primary concern in clinical application that biological agents suffer from, i.e., low application efficiency. Although genetical engineering and physiochemical engineering provides abundant means to address it. They are confronted with their individual sufferings. Typically, genetical engineering inevitably increase the risk that off‐target poses uncontrollable and unexpected gene pollution and symptoms when integrating multiple genes into one bacterial. Although the marriage of biological system and nonbiological systems represented by physiochemically‐engineered bacteria has no gene instability‐arised biosafety concern, the unresolved and disputed material safety or degradation remain a risk factor to bring about the diagnostic failure of physiochemically‐engineered bacteria.
3. Clinical translation has a long way, and no approval in clinics derives from the fear of bacteria slow down this process even though there are evidences on safety and treatment efficacy of engineered oncolytic bacteria. To remove the fear, more comprehensive and systematic safety evaluations need to be carried on, and especially the specific and precisely‐controllable expansion and replication in targeted zone should be satisfied. Additionally, the innate immune response and cytokine storm should be attenuated to ensure the treatment safety.
4. In future developments, engineered bacteria can be tailored to meet individual patient needs based on modified bacterial composition and genes and tumor markers to carry out personalized disease therapy. It is worth mentioning that the use of physiochemically‐ and genetically‐engineered bacteria for clinical translation requires more practical and theoretical application basis.
5. Although there are some combined therapy protocols based on engineered bacteria, the complicated microenvironment and pathogenesis of one lesion means that there are many supplementary mechanisms that render such a lesion intractable and highly tolerant. Regarding this, more combined projects are necessary, e.g., two or more physiochemical engineering combinations, physiochemical and genetical engineering combinations, two or more genetical engineering combination, etc.
6. Personalized application is often preferable in the development of drug treatment for clinical patients. It is highly desirable to combine physiochemically‐engineered and genetically‐engineered technologies to design precise medical treatment protocols.
7. In light of gut microbiota correlate with many diseases and multiple organ or tissue homeostasis, more application fields can be explored.

## Conflict of Interest

The authors declare no conflict of interest.

## Author Contributions

X.L., R.J., and H.C. contributed equally to this work. K.Z. proposed this theme, organized the skeleton, provided the raw materials and selected the cited references. X.L., R.J., H.C., X.Y. and K.Z. re‐organized figures and wrote the manuscript. K.Z. wrote this manuscript, supervised and supported the project. All authors commented on this manuscript.
